# Molecular Characterization and Immuno-Reactivity Patterns of a Novel *Plasmodium falciparum* Armadillo-Type Repeat Protein, PfATRP

**DOI:** 10.3389/fcimb.2020.00114

**Published:** 2020-03-20

**Authors:** Emmanuel Amlabu, Philip Ilani, Grace Opoku, Prince B. Nyarko, Evelyn Quansah, Laty G. Thiam, Manfred Anim, Reuben Ayivor-Djanie, Ojo-ajogu Akuh, Henrietta Mensah-Brown, Julian C. Rayner, Gordon A. Awandare

**Affiliations:** ^1^West African Center for Cell Biology of Infectious Pathogens, University of Ghana, Accra, Ghana; ^2^Department of Biochemistry, Kogi State University, Anyigba, Nigeria; ^3^Department of Biomedical Sciences, SBBS, University of Health and Allied Sciences, Ho, Ghana; ^4^Cambridge Institute for Medical Research, University of Cambridge, Cambridge, United Kingdom; ^5^Department of Biochemistry, Cell and Molecular Biology, College of Basic and Applied Sciences, University of Ghana, Accra, Ghana

**Keywords:** PfATRP, recombinant protein, immunolocalization, immunoreactivity, serosurveilance

## Abstract

Nearly half of the genes in the *Plasmodium falciparum* genome have not yet been functionally investigated. We used homology-based structural modeling to identify multiple copies of Armadillo repeats within one uncharacterized gene expressed during the intraerythrocytic stages, PF3D7_0410600, subsequently referred to as *P. falciparum* Armadillo-Type Repeat Protein (PfATRP). Soluble recombinant PfATRP was expressed in a bacterial expression system, purified to apparent homogeneity and the identity of the recombinant PfATRP was confirmed by mass spectrometry. Affinity-purified α-PfATRP rabbit antibodies specifically recognized the recombinant protein. Immunofluorescence assays revealed that α-PfATRP rabbit antibodies reacted with *P. falciparum* schizonts. Anti-PfATRP antibody exhibited peripheral staining patterns around the merozoites. Given the localization of PfATRP in merozoites, we tested for an egress phenotype during schizont arrest assays and demonstrated that native PfATRP is inaccessible on the surface of merozoites in intact schizonts. Dual immunofluorescence assays with markers for the inner membrane complex (IMC) and microtubules suggest partial colocalization in both asexual and sexual stage parasites. Using the soluble recombinant PfATRP in a screen of plasma samples revealed that malaria-infected children have naturally acquired PfATRP-specific antibodies.

## Introduction

Malaria is a major global health problem that poses a threat to half of the world's population. Globally, 272 000 malaria deaths were estimated to be in children aged under 5 years (WHO, 2019). The intra-erythrocytic stage of the parasite life cycle is a key target for vaccine and drug development, since all clinical symptoms of malaria occur during this stage. Erythrocyte invasion by the malaria parasite is a complicated and highly coordinated process that involves attachment and penetration mediated by a sophisticated network of parasite proteins discharged from two apical secretory organelles, the rhoptries and micronemes (Cowman et al., 2017). Another key invasion-associated organelle is the inner membrane complex (IMC), which contributes to the maintenance of cell morphology and rigidity (Aikawa et al., 1981; Meszoely et al., 1987; Kono et al., 2012), but also plays a role in motility and invasion, by acting as an anchor for the actin-myosin motor that provides the pre-requisite force necessary for invasion processes (Soldati et al., 2004; Baum et al., 2006, 2008; Jones et al., 2006; Yeoman et al., 2011).

The Armadillo Repeat Motif (ARM) is present in proteins across the eukaryotic lineage and has been associated with protein-protein interactions such as bridging the cytoplasmic domains of cadherins to α-catenin and the actin cytoskeleton (McCrea et al., 1991; Hülsken et al., 1994). In *Plasmodium* and other related parasites, ARM-containing proteins have been shown to have multiple functions including DNA-binding (Mitra et al., 2016), apical positioning of the rhoptry organelle, a pre-requisite for host cell invasion (Mueller et al., 2013), clustering of rhoptry organelles (Mueller et al., 2016), cell signaling, cytoskeletal organization, gene regulation (Coates, 2003; Tewari et al., 2010) and recently, IMC formation (Absalon et al., 2016). PF3D7_0410600-interacting partner proteins have been discovered using a proteome-wide yeast-2-hybrid screen approach (LaCount et al., 2005). Interestingly, one of the PF3D7_0410600 binding partners; 14-3-3 is a hub protein that plays important roles in many regulatory processes including mitogenic signal transduction, apoptotic cell death, cell cycle control, and protein localization (Fu et al., 2000; Schechtman et al., 2001; Assossou et al., 2003). In *T. gondii*, 14-3-3 protein has shown potential as a vaccine candidate against toxoplasmosis (Meng et al., 2012) and the protein has been implicated in the mechanism developed by parasites to stimulate host immune responses. Considering the future prospects in taking advantage of protein-protein interactions for the development of better diagnostic tools for malaria infection, we sought to functionally characterize PF3D7_0410600 protein especially that it may play an important role in parasite-specific processes or could be a potential biomarker.

In this report, we identified a novel ***P***. ***f**alciparum*, **A**rmadillo-**T**ype **R**epeat **P**rotein (PfATRP: PF3D7_0410600/PFD0525w) based on its gene expression profile and potential function in protein-protein interactions. We hypothesized that PfATRP may be involved in merozoite egress or invasion and tested for an egress phenotype during schizont arrest assays and demonstrated that native PfATRP is inaccessible on the surface of merozoites in intact schizonts. We also determined the subcellular localization of the protein and measured its antibody levels in children exposed to varying intensities of *P. falciparum* infection in Ghana.

## Results

### PfATRP Structural Characteristics and Time-Resolved Expression Analysis

**Figure 1:**
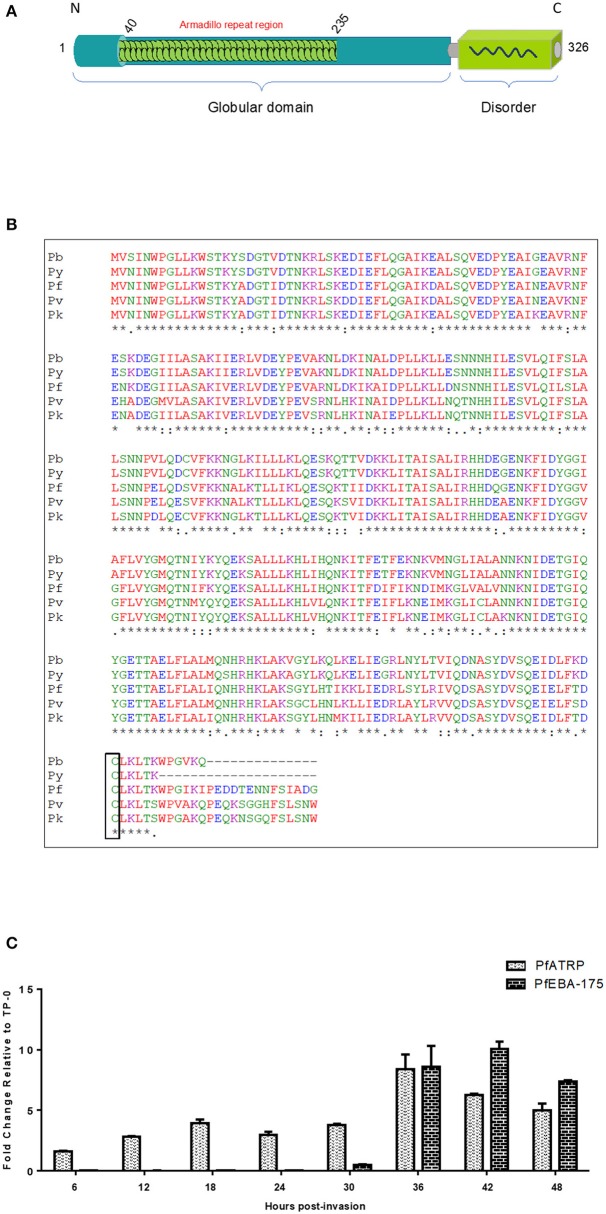
PfATRP structural characteristics and time-resolved expression analysis. **(A)** Domain architecture of PfATRP showing the features of the full-length protein. **(B)** Clustal 0 (1.2.4) multiple sequence alignment (72% similarity) shows that PfATRP is conserved across different Plasmodium species orthologs. **(C)** Time-resolved qRT-PCR expression profile of PfATRP asexual parasite stages. The gene transcript levels for PfATRP was analyzed with PfEBA-175 as control at eight (8) different time-points (6 h interval).

The PfATRP gene (PF3D7_0410600) is a 3-exon gene located on chromosome 4 and encodes a 326-amino acid protein with a predicted molecular weight of 32 kDa. Plasmodium database (PlasmoDB) shows that the protein lacks a recognizable signal peptide and transmembrane domain (Figure 1A). Also, expert protein analysis system (ExPASy), (Gasteiger et al., 2005) indicates that the protein does not possess any myristoylation or acetylation signal (Figure 1A). PfATRP is evolutionarily conserved across rodent and primate *Plasmodium* species (Figure 1B) and all orthologs have a positionally-conserved cysteine residue at the C-terminal end of the protein. PfATRP structure was predicted by homology modeling using both Phyre 2 and I-TASSER. Homology modeling by Phyre 2 predicts that PfATRP exhibits structural features of the β-catenin family that harbor armadillo repeats (Figure S1A). Similar modeling using I-TASSER, revealed that PfATRP shares structural similarities with importin-β (Figure S1B). These results from the two protein prediction servers were consistent, since armadillo repeats are known to adopt similar structural conformation in β-catenin and importins (Lee et al., 2000; Koike et al., 2004). Overall, PfATRP seems to be globular in nature with a very short disordered region at the C-terminus. Also, PfATRP possesses an Armadillo Repeat Motif (ARM), a characteristic feature of the β-Catenin family of proteins. These motifs are also potential docking sites for protein-protein interactions (Coates, 2003; Tewari et al., 2010), and ARM containing proteins have been shown to be associated with invasion-related organelles in *P. falciparum* merozoites.

To confirm when *PfATRP* was expressed, we extracted RNA from multiple time points across the *P. falciparum* spaced six (6) h apart. Time-resolved qRT-PCR revealed that *PfATRP* is expressed through all the time-points during the asexual stage. However, *PfATRP* and *PfEBA-175* (Bozdech et al., 2003) peaks maximally at between 36–42 h post-invasion, respectively (Figure 1C), suggesting that *PfATRP* may also play a role in egress or invasion. These data are in keeping with previously published RNAseq data (Otto et al., 2010), which also confirm a late-stage expression profile for *PfATRP*, and suggests a role in late-stage development, such as merozoite generation, egress, or erythrocyte invasion.

### Production of Recombinant PfATRP and Antibody Generation

**Figure 2:**
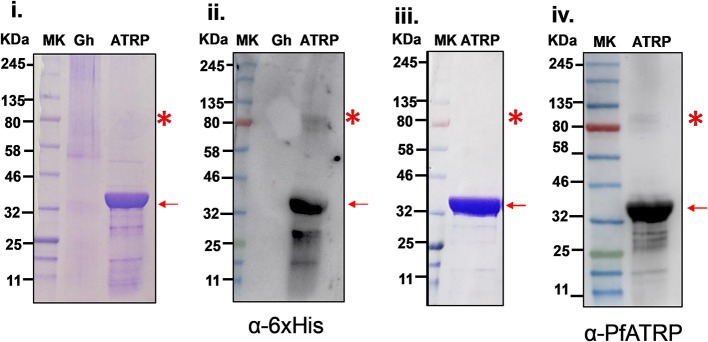
Production of recombinant PfATRP and immunoblotting. **(i)** Coomassie-stained gel shows the recombinant PfATRP (35kDa) that was purified on Ni-NTA column. **(ii)** The recombinant protein was detected by immunoblotting using α-Histidine mouse monoclonal antibody. **(iii-iv)** Coomassie-stained gel shows the recombinant PfATRP (35 kDa) which was also detected by immunoblotting using α-PfATRP rabbit antibody. Gh: solubilized erythrocyte ghost membrane proteins. Protein samples were ran along side with color, pre-stained protein standard (P7712S) broad range (11–245 kDa).

Recombinant PfATRP expressed in bacterial system produced a dominant 35 kDa protein species consistent with its theoretical molecular weight determined based on its codon-optimized sequence (Figures S2, S3A) Recombinant PfATRP was purified under non-denaturing conditions using immobilized metal affinity column that resulted in the enrichment of both 35 kDa and a second, much fainter, ~80 kDa recombinant PfATRP species (Figure S3B). Anti-6xHistidine tag mouse monoclonal antibody detected the 35 kDa and ~80 kDa species of PfATRP during immunoblotting of the nickel-nitrilotriacetic acid (Ni-NTA) purified recombinant protein (Figures 2i,ii). The Ni-NTA eluates were pooled, concentrated using 10 kDa cutoff centricons and further purified on size exclusion chromatography (SEC) column (Figure S3B). Using the codon optimized sequences (Figures S2i-iv), the control antigens, PF3D7_1404900 (30 kDa), PfMSP7 (50 kDa), and PF3D7_0308300 (40 kDa) were expressed and purified to apparent homogeneity (Figure S4). The identities of the control recombinant proteins were confirmed by immunoblotting using α-6xHistidine mouse monoclonal antibody (Figure S4).

The identity of the recombinant PfATRP was further confirmed by Liquid chromatography-Mass Spectrometry (LC-MS), (Table S1) and recombinant PfATRP was immunized in rabbits following a prime-double booster immunization regimen and antibodies were generated by Biobasic, Canada. To explore the identity of the two bands detected by α-6xHistidine mouse monoclonal antibody (Figures 2i,ii), immunoblotting was performed for the SEC-purified PfATRP. PfATRP was specifically detected during immunoblotting using α-PfATRP rabbit antibody (Figures 2iii,iv) which indicated that SEC removed most of the ~80 kDa recombinant PfATRP species.

### Anti-PfATRP Rabbit Antibodies Reacts With *Plasmodium falciparum* Mature Schizonts

**Figure 3:**
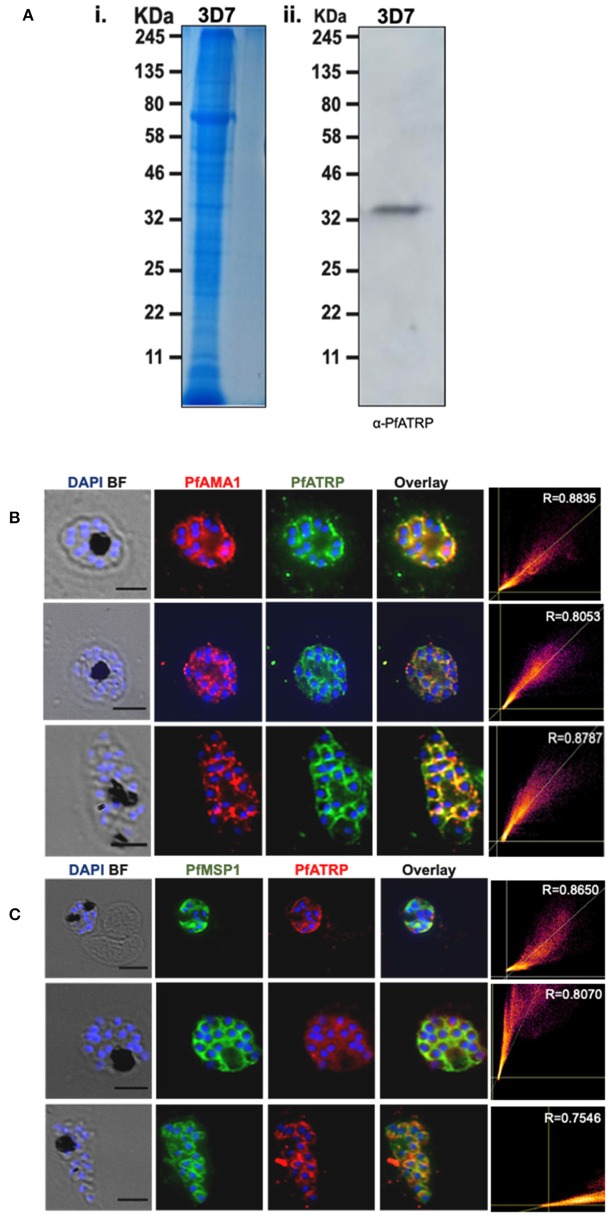
Anti-PfATRP rabbit antibody reacts with *P. falciparum* matured schizonts. **(A)** Commassie stained gel showing the profile of proteins in 3D7 detergent-treated schizont lysates (i). Anti-PfATRP rabbit antibody detects native PfATRP in 3D7 detergent-treated schizonts lysates by immunoblotting (ii). **(B)** Dual IFA staining performed under permeabilized conditions using segmenting schizonts showed that α-PfAMA1 mouse antibody (red); (1:100) and α-PfATRP rabbit antibody (green); 1:100 labeled schizonts. **(C)** α-PfATRP rabbit antibody (red); (1:100) and α-PfMSP1 mouse antibody (green); (1:100) labeled schizonts. The nuclei were stained with DAPI. Black lines on IFA images represent scale bar. R signifies the colocalization coefficient.

To determine if α-PfATRP rabbit antibody specifically reacts with native PfATRP in schizont lysates, we performed immunoblotting using 3D7 detergent-treated schizont lysates. Coomassie stained gel shows the profile of proteins in the parasite lysates which was probed with α-PfATRP rabbit antibody that specifically recognized the 35 kDa protein band corresponding to the expected molecular weight for native PfATRP (Figures 3Ai,ii). The detection of a prominent signal during immunoblotting for native PfATRP excludes the possibility of any off-target reactivity of α-PfATRP rabbit antibody. Next, we performed permeabilized dual immunofluorescence assays (IFAs) during which we tested whether α-PfAMA1 (micronemal marker) or α-PfMSP 1 mouse and α-PfATRP rabbit antibodies reacted with segmenting schizonts. These showed that α-PfAMA1 mouse and α-PfATRP rabbit antibodies labeled the periphery of schizonts with dotty staining patterns suggesting an overlap of PfATRP with PfAMA1 (Figure 3B). We performed co-staining of α-PfATRP rabbit and α-PfMSP1 mouse antibodies in asexual stage parasites and this indicated that PfATRP is not likely localized on the merozoite surface (Figure 3C and Figure S4). Similarly, dual IFAs were performed for gametocytes using α-PfATRP rabbit antibody and α-Pfs48/45 mouse antibody) which showed that PfATRP is not localized on the surface of gametocytes (Figure S5).

### PfATRP Is Not Localized on the Parasite Surface

**Figure 4:**
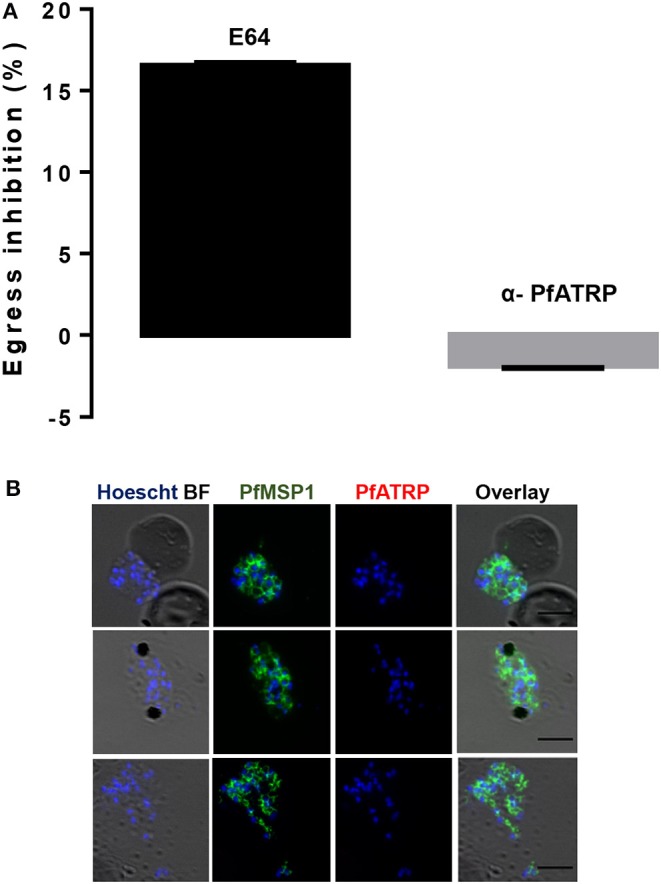
PfATRP is not localized on the parasite surface. **(A)** Egress assays were performed with 3D7 parasites using α-PfATRP rabbit antibody and E64 was used as control, showing that α-PfATRP rabbit antibody does not inhibit schizont egress or reinvasion of erythrocytes by merozoites. **(B)** α-PfMSP1 mouse antibody stained the surface of merozoites in non-permeabilized schizonts during in-solution IFA, but no staining was observed for α-PfATRP rabbit antibody. The nuclei were stained with Hoescht. Black lines on IFA images represent scale bar.

Given ATRP location in developing merozoites, one possibility is that it may be involved in merozoite egress from infected erythrocytes. We tested for an egress phenotype using the schizont arrest assay that is based on the permeability of parasitized erythrocytes to macromolecules, including antibodies, at the later stages of schizogony (Ahlborg et al., 1996; Goodyer et al., 1997; Bergmann-Leitner et al., 2009; Raj et al., 2014). We showed only ~15% egress inhibition by 10 μM E64, a cysteine protease that is known to arrest the egress of merozoites and subsequently resulted in low parasitemia upon reinvasion of erythrocytes. This was because we used segmented schizonts and this was to ensure antibodies got access to the parasites within the erythrocytes. Besides, it was reported previously (Hill et al., 2012) that segmented schizonts are less affected by E64 treatment. Anti-PfATRP rabbit antibody at 100 μg/mL did not block merozoite egress or subsequent reinvasion of new erythrocytes (Figure 4A). This suggests that PfATRP may not be accessible on the parasite surface.

The IMC is the only known organelle consistent with a peripheral staining pattern around the merozoite that is not surface accessible. Proteins localizing to the IMC, a cytoskeleton compartment that sits immediately under the merozoite surface, can appear largely peripheral in IFAs. To distinguish between surface and IMC staining, we performed non-permeabilized in-solution IFA's during which we tested whether both α-PfATRP rabbit and α-PfMSP1 mouse antibodies reacted with the merozoite surface. Non-permeablized in-solution IFA's showed that α-PfMSP1 mouse antibody labeled the surfaces of merozoites in intact segmented schizonts (Figure 4B), but α-PfATRP rabbit antibodies did not label the merozoite surfaces (Figure 4B). Altogether, this provides two lines of evidence that PfATRP is not accessible on merozoite surface unlike PfMSP1 (Das et al., 2015) and PfAMA1 (Douglas et al., 2013) that are surface accessible as reported previously. PfATRP is suggested by the PlasmoGEM and piggyBac studies to be essential for blood stage growth and it is most highly expressed in schizonts. Therefore, it is likely that PfATRP has an essential function in schizonts that could be through a non-surface exposed mechanism required for merozoite development, egress or invasion.

### PfATRP Probably Exhibits IMC and Microtubular Localization Patterns

**Figure 5:**
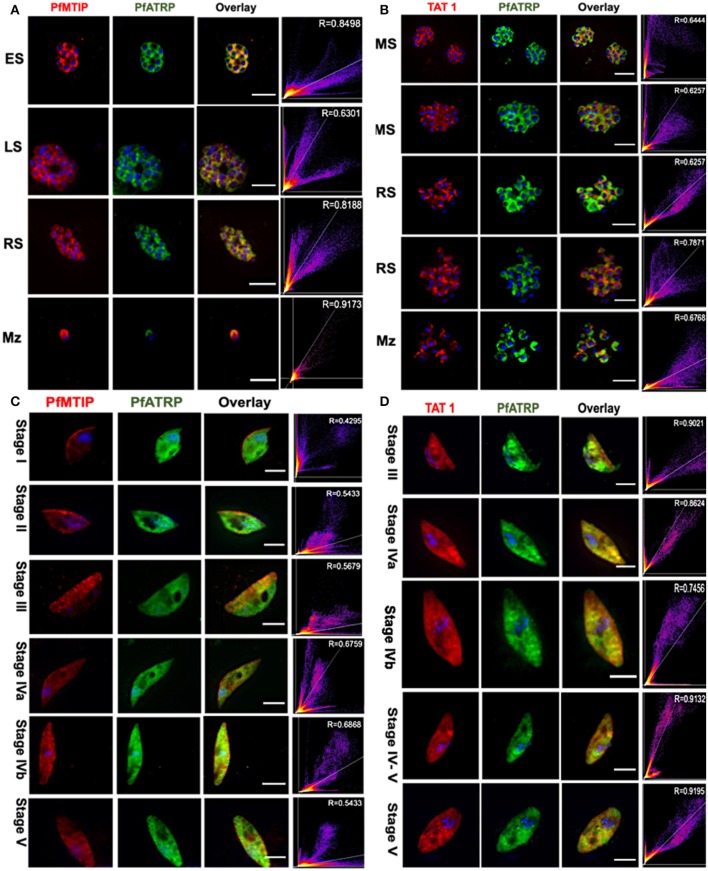
PfATRP probably exhibits IMC and microtubular localization pattern. Dual IFAs were performed for both asexual and sexual parasite stages using markers for different subcellular organelles. **(A)** Slides were prepared for asexual stage parasites (early schizonts (ES); late schizonts (LS); rupturing schizonts (RS) and free merozoites (Mz), and probed with α-PfATRP rabbit antibody (green); (1:100) and α-PfMTIP rat antibody (1:100); (red) which showed that PfATRP partly colocalized with PfMTIP in the inner membrane complex. **(B)** Similarly, dual IFAs were performed for matured schizonts (MS), rupturing schizonts (RS) and free merozoites (Mz). α-TAT1 mouse antibody (red); (1:10), the marker for microtubules and α-PfATRP rabbit antibody (green) (1:100) did not colocalize. **(C)** Gametocytes (stages I-V) were co-stained with α-PfMTIP rat antibody (red), (1:100) and α-PfATRP rabbit antibody (green), (1:100); and a restricted colocalization pattern was observed. **(D)** Co-staining of gametocytes with α-TAT-1 mouse antibody (red), (1:10) and α-PfATRP rabbit antibody (green); (1:100) showed colocalization which was largely cytoplasmic. White lines on IFA images represent scale bar. R signifies the colocalization coefficient.

To determine whether PfATRP is localized to the IMC in asexual stage parasites, we performed dual immunofluorescence staining of early-, late-, rupturing schizonts and free merozoites using α-PfATRP rabbit antibody with the IMC marker, α-Myosin A-tail Interacting Protein (PfMTIP) rat antibody (Dearnley et al., 2012). We observed that α-PfATRP rabbit antibody partly colocalizes with α-PfMTIP rat antibody (Figure 5A) indicating that a sub-population of PfATRP is localized to the IMC. In early-, late-, rupturing-schizonts and free merozoites, there was no overlap of α-PfATRP rabbit and α-Tubulin Acetyltransferase 1 (TAT1) mouse antibody. This suggests that PfATRP might not be associated with microtubules during asexual stages (Figure 5B).

PfATRP expression has also been reported in gametocytes (Tao et al., 2014) and based on annotated gene ontology component and predicted gene ontology function, the protein has been linked with microtubule motor activity which is associated with the dynein complex. In gametocytes, α-PfATRP rabbit antibody labeled gametocytes and a restricted colocalization pattern with α-PfMTIP rat antibody was observed (Figure 5C). Contrary to the observation in asexual stage parasites, α-PfATRP rabbit antibodies colocalizes with TAT1 (McRobert et al., 2008) that recognizes all tubulin isoforms suggesting an association with the microtubular network in gametocytes (Figure 5D). This is consistent with the crucial role of another ARM protein (PF16) which is expressed in male gamete flagellum, where it maintains the correct microtubule structure in the central apparatus of the axoneme (Straschil et al., 2010). Therefore, it is conceivable that the observed disparity in PfATRP subcellular localization during the asexual and sexual parasite stages could be developmentally regulated.

### PfATRP Is Recognized by Antibodies From Individuals Naturally Exposed to *P. falciparum* Malaria

Malaria transmission intensity has been measured by the entomological inoculation rates and was highest in Kintampo (>250 infective bites/person per year), followed by Navrongo (<250 infective bites/person per year), and lowest in Accra (<50 infective bites/person per year)(Klinkenberg et al., 2008; Kasasa et al., 2013). The Samples used in this study were collected during the rainy seasons at the respective study sites between September 2011 and September 2013. The immunoreactivity of PfATRP in comparison with three other parasite antigens (PF3D7_1404900, PfMSP7 and PF3D7_0308300) were evaluated using plasma samples from malaria-infected children resident in three endemic areas within Ghana with varying transmission intensities (Klinkenberg et al., 2008; Kasasa et al., 2013).

PF3D7_1404900 (PF14_0046) is a novel protein that was localized at the parasite periphery typical of parasite plasma membrane (PPM), parasitophorous vacuole (PV) or parasitophorous vacuolar membrane (PVM) by green fluorescent protein (GFP)-tagging approach (Heiber et al., 2013). More recently, GFP-tagged PF3D7_1404900 protein was localized in merozoites revealing a cytosolic staining pattern with accumulation at the apical pole (Wilcke, 2018). *Plasmodium falciparum* Merozoite Surface Protein 7 (PfMSP7) is involved in erythrocyte invasion and the protein is currently under study as a potential malaria vaccine candidate (Raj et al., 2014). PF3D7_0308300 is another novel protein associated with merozoite invasion and was localized to the parasite surface (Hu et al., 2010) by expressing the GFP-tagged protein ectopically in *P. falciparum* (Treeck et al., 2006). Interestingly, PF3D7_0308300 protein is one of the *P. falciparum* antigens on the Malian protein microarray that met the inclusion criteria during the assessment of anti-malaria antibody responses (Helb, 2015).

**Figure 6:**
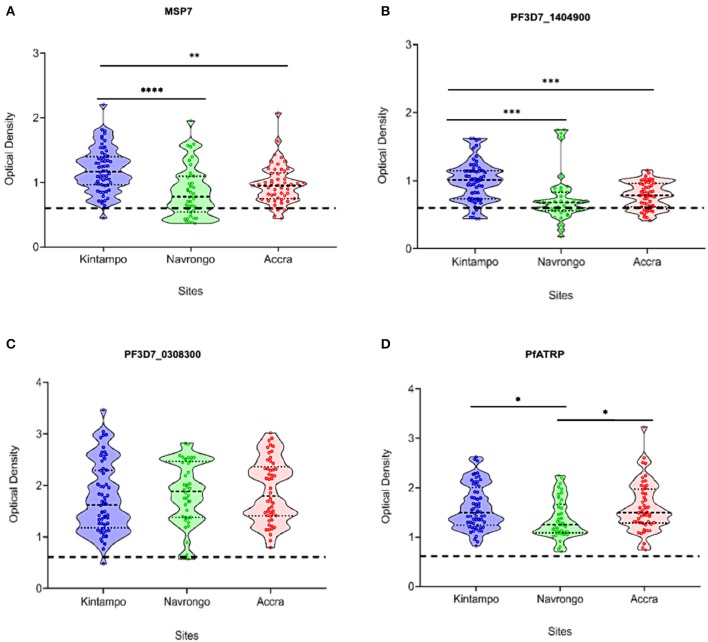
PfATRP is recognized by antibodies from individuals naturally exposed to *P. falciparum* malaria**. (A–D)** Violin plots showing the levels of antibody responses against the four different antigens (PfMSP7, PF3D7_1404900, PF3D7_0308300, and PfATRP) across areas of varying malaria transmission intensity (Kintampo>Navrongo>Accra). Statistical analyses were performed using One-way ANOVA and where significant, Tukey's multiple comparison test was used to compute for pairewise comparisons. All graphs were performed on GraphPad Prism v. 8.01. The black dotted-lines in the graphs depict the cut-off of seropositivity defined using the mean + 3 standard deviations of the OD values obtained for the naïve control. Significant statistical differences were shown. *p = 0.03, **p = 0.002, ***p < 0.001, ****p < 0.0001. ns: non-significant. Sample sizes: Kintampo (*n* = 60); Navrongo (*n* = 44); Accra (*n* = 57) and naïve control (*n* = 6). The ELISA data shown in this figure is a representation of the means of two independent experiments.

The basis for the use of these antigens with known subcellular localization as comparators for PfATRP is to determine how the intracellular localization of these antigens in released merozoites modulates their respective plasma immunoreactivity patterns (Dreyer et al., 2012). We assessed the levels of antibody responses against four different *P. falciparum* merozoite antigens (PfMSP7, PF3D7_0308300, PF3D7_1404900, and PfATRP) across areas of varying malaria transmission intensity (Kintampo>Navrongo>Accra); (Klinkenberg et al., 2008; Kasasa et al., 2013). The data showed that the immunoreactivity patterns for both PfMSP7 and PF3D7_1404900 varied significantly with transmission intensity when comparisons were made between Kintampo and Navrongo, Kintampo and Accra (Figures 6A,B). However, there were no significant differences in the response patterns for PfMSP7 and PF3D7_1404900 between Navrongo and Accra (Figures 6A,B). Also, there was no discernable transmission intensity effect on the levels of PF3D7_030800 and PfATRP plasma antibodies (Figures 6C,D) which indicates that both antigens are not likely to be considered as a potential biomarkers.

Consequently, the observed differences in the response patterns for these antigens is consistent with the proposition that differences in the rate of antibody acquisition to merozoite antigens differ due to differences in immunogenicity and protein subcellular localization (McCallum et al., 2017). Moreover, differences in the responses between the antigens could also be linked to the fact that different *P. falciparum* antigens elicit antibody responses with different magnitudes and kinetics (Elliott et al., 2014; Stanisic et al., 2015).

### PfATRP Human Antibody Response Does Not Correlate Significantly With Age

**Figure 7:**
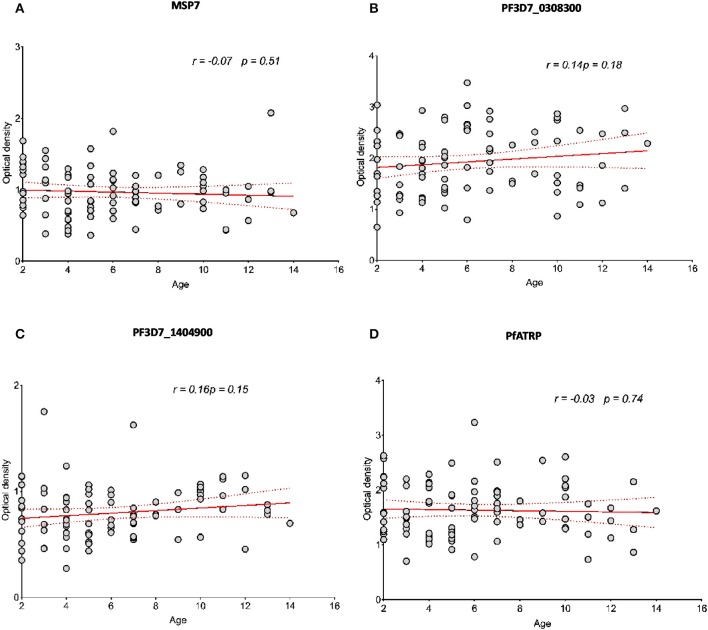
PfATRP human antibody response does not correlate significantly with age. **(A–D)** The scattered plots represents the pooled and age-matched data analysis for the antibody response patterns across the three malaria endemic sites for all antigens tested. The red lines depict the linear regression lines (plain lines) with their associated 95% confidence intervals (dotted lines). The Pearson correlation test was used to compute the coefficients of correlation between the measured variables. All statistical analyses were performed using GraphPad Prism v. 8.01.

In order to evaluate the applicability of α-PfATRP human antibody during serological screening of a population, we used age-stratified plasma samples across all sites for the analysis of PfMSP7, PF3D7_0308300, PF3D7_1404900, and PfATRP responses. The data showed that none of these antigens tested correlated significantly with age (Figures 7A–D). Furthermore, we substantiated the ELISA-based analysis for PfATRP response by performing immunoblotting experiments. The non-reactive control did not detect recombinant PfATRP (Figure S6i) while the reactive control plasma from malaria-exposed healthy adults (Figure S6ii) and the individual immunoreactive pools from children across the three malaria endemic sites detected recombinant PfATRP (Figures S6iii-v). Thus, our data on the immunoreactivity profiles of PfATRP presents it as an immunogenic antigen.

## Discussion

The availability of published *P. falciparum* genome (Gardner et al., 2002), transcriptome (Bozdech et al., 2003) and proteomic datasets (Bowyer et al., 2011) makes the identification and functional characterization of novel parasite proteins feasible, yet most *P. falciparum* genes/proteins remain to be characterized functionally. Two recent large-scale genetic screens for blood-stage phenotypes in *P. berghei* and *P. falciparum* have added a first layer of functional data for most genes in the genome (Schwach et al., 2015), but while this represents a significant step forward, detailed localization, and biochemical studies of individual proteins will still be required to illuminate their functions. We identified a novel *P*. *falciparum*, Armadillo-Type Repeat Protein (PfATRP: PF3D7_0410600/PFD0525w) using bioinformatics portals, which revealed some predicted structural characteristics of the protein. The encoding gene, *PfATRP*, was suggested to be essential for blood-stage parasite growth in a recent *piggyBac* transposon saturation-level mutagenesis screen (Zhang et al., 2018). Generally, ARM proteins are known to be versatile in their functions, and the classification of ARM proteins has been quite challenging given that some of the current annotations of armadillo repeats are incomplete or may be incorrect (Gul et al., 2017), due in part to difficulties in distinguishing between armadillo repeat types and the high similarity with Huntingtin, elongation factor 3, protein phosphatase 2A, and the yeast kinase TOR1 (HEAT) repeats (Kippert and Gerloff, 2009). Detailed functional phenotyping of individual ARM proteins is therefore important.

We used α-PfATRP rabbit antibody during schizont arrest assays/in-solution or non-permeabilized IFAs and showed that PfATRP is not localized on the merozoite surface. Colocalization analysis performed during permeabilized IFAs in both asexual and sexual stages suggest that PfATRP may exhibit a dynamic subcellular localization. The identification of PfATRP that lacks membrane attachment motifs as a likely component of the IMC/microtubules in the parasite was unexpected because previously characterized ARM-containing proteins (Absalon et al., 2016; Jacot et al., 2016; Mitra et al., 2016) were all localized to the apical pole. However, a previous report supports the possibility that ARM repeats could mediate membrane association (Drechsel et al., 2010). Therefore, immuno-electron microscopy analysis will be required to determine the precise subcellular localization of PfATRP in the parasite.

PfATRP and other ARM proteins belong to the β-catenin family that interacts with dynein and appears to tether microtubules at adherens junctions in epithelial cells (Ligon et al., 2001). In gametocytes, we observed colocalization of PfATRP with TAT1 which supports an association with microtubules. Hence, the localization of PfATRP in the IMC and its associated microtubules may represent the poorly described subcellular organelle interplay in the parasite. Although, it is noteworthy that the IMC-microtubular interplay may have resulted during lateral expansion of the IMC around the girth of the parasite when it associates with microtubules (Schneider et al., 2017).

The IMC is a cisternal compartment that is assembled under the plasma membrane of *Plasmodium* parasites, in merozoites, sporozoites, ookinetes, and gametocytes (Schneider et al., 2017). The IMC plays an important structural role in the cellular remodeling events associated with gametocyte elongation (Dearnley et al., 2012). This is consistent with studies that indicated the roles which specific IMC proteins play in cell morphology (Tremp and Dessens, 2011). During gametocyte development, elongation is driven by a network of microtubules that assemble under the IMC (Schneider et al., 2017). Importantly, the gametocyte IMC has a stage-specific function that may involve a poorly defined set of proteins (Schneider et al., 2017) which have been classified based on their structural features into alveolins, non-alveolins, and multi-transmembrane proteins (Kono et al., 2012). However, a member of the glideosome assembly, PfGAP50 is known to be recruited to the periphery of gametocytes and appears to be coordinated with the laying down of microtubules (Dearnley et al., 2012). There are a number of parasite proteins that are recruited to the IMC via protein-protein interactions (Ramirez and Lowe, 2009; Kono et al., 2012; Schneider et al., 2017). Although putative PfATRP-interacting partner proteins have been reported previously by proteome-wide yeast-2-hybrid screens (LaCount et al., 2005), the relevance of this in the context of PfATRP membrane attachment has not been evaluated. PfATRP may not be palmitoylated based on previous studies on *P. falciparum* Palmitome (Jones et al., 2012) which leaves avenues for future studies since other parasite proteins have been shown to deploy lipid modification mechanisms for membrane attachment (Wetzel et al., 2015).

The identification of Plasmodium species-specific biomarkers is important in areas where multi-species infections occur. This provides information that will guide the evaluation of control measures that targets a single species, which might impact on the transmission and immunological profiles of other co-endemic species (Herman et al., 2018). The understanding of immunity to infections can be deduced from studies on human serological responses to different Plasmodium species. There is increasing interest in the use of antibodies specific for merozoite antigens as serological biomarkers of Plasmodium exposure or as biomarkers of protective immunity (Osier et al., 2014; Helb et al., 2015; Stanisic et al., 2015; van den Hoogen L. L et al., 2019). Although, immuno-epidemiological studies have recorded repeated conflicting data on responses to the same antigen in different areas (Riley et al., 1992; Høgh et al., 1995; Al-Yaman et al., 1996; Branch et al., 1998; Cavanagh et al., 2004), large-scale sero-epidemiological analysis performed on a microarray platform has always provided useful information. A previous microarray-based analysis of plasma samples revealed that antibodies to intracellular proteins were better viewed as biomarkers of past infection or indicators of enhanced parasite killing in protected individuals, and not as evidence for vaccine potential (Crompton et al., 2010). PfATRP may be an intracellular antigen that is immunogenic but its potential application as a biomarker is not supported by the data in this study. Therefore, we presume that the naturally acquired, α-PfATRP human antibody responses observed in malaria exposed children could be attributed to high biomass of *P. falciparum* parasites during advanced infections that results in the lysis of non-invasive merozoites which expose their contents to the immune system.

Altogether, this work presents the first cell biological and immunological assessment of PfATRP that presents new opportunities in targeting protein localization for anti-infective therapy. Further functional investigation on the structural determinant mediating the recruitment of PfATRP to membrane localization is required.

## Materials and Methods

### PfATRP Domain Identification and Homology Modeling

The amino acid sequence of PfATRP was submitted to the Eukaryotic Linear Motif (ELM) portal as described previously (Dinkel et al., 2015). This platform analyzes user-submitted protein sequences by scanning for matches to structural motifs that are already curated in the database. A predicted 3D-model for full length PfATRP was determined using both Phyre 2 and I-TASSER protein homology modeling platform for structural predictions (Roy et al., 2010; Kelley et al., 2015).

### Parasite Culture and Synchronization

*P. falciparum* strains (3D7 and NF54) were cultured in normal human O^+^ erythrocytes based on the methods described previously (Jensen and Trager, 1978). The parasites were tightly synchronized by routine sorbitol treatments and Percoll-alanine gradient centrifugation (Kanaani and Ginsburg, 1989; Awandare et al., 2018).

### Quantitative RT-PCR Analysis

Total RNA was extracted from 3D7 parasite pellets at 8-h intervals using the phenol-chloroform method after homogenization with TRIzol Reagent (Ambion/Life Technologies, Carlsbad, California). Quantitative mRNA transcript levels for the control, Erythrocyte Binding Antigen 175 (EBA-175) was determined using the primer set (forward primer (FP): TTCGTGATGAGTGGTGGAAA and reverse primer (RP): GGCAATAATCATCACCCCATT) (Lopaticki et al., 2011). PfATRP transcripts levels were also determined using the designed primers (FP: ACGAAATATGCAGACGGGACT and RP: CGAAGTTACGAACGGCTTCATT). Analysis was performed on a Quant Studio 5, Real-Time PCR system (Applied Biosystems) using the Luna Universal, One-Step RT-qPCR Kit (New England Biolabs, Inc.) following the manufacturer's protocol. The mRNA transcript levels were normalized to the expression of the 60S ribosomal protein L18 (2^−Δ*Ct*^).

### Gene Synthesis and Sub-cloning

A codon harmonized, full-length gene of 981 bp, encoding 326 amino acids (Met-1 to Gly-326) of PfATRP from *P. falciparum* with a C-terminal Hexa-histidine (6x His) tag was synthesized and sub-cloned by Bio Basic (Canada) into a T7 promoter *Escherichia coli* (*E. coli*) expression vector (pET-24b) with Nde1 and Xho1 restriction sites to obtain PfATRP plasmid for enhanced expression in *E. coli*. Also codon optimized genes for PF3D7_1404900 (84-894 bp), PfMSP7 (93-1056 bp), and PF3D7_0308300 (1-1014 bp), encoding 269 amino acids (Asn-29 to Leu-297); 351 amino acids (Asn-32 to Met-351) and 337 amino acids (Met-1 to Asn-337), respectively were synthesized and subcloned in pET-24b vector with Nde1 and Xho1 restriction sites for expression in *E. coli*.

### Recombinant Protein Production

PfATRP, plasmid was transformed into BL21-CodonPlus (DE3)-RIPL *E. coli* competent cell and cultures were induced at an optical density of 0.6 with 1 mM Isopropyl β-D-1-thiogalactopyranoside (IPTG) at 25°C for 12–18 h. The expressed recombinant protein was purified by immobilized metal affinity chromatography (IMAC) using Ni-NTA resin (Qiagen, USA). The purified protein was buffer-exchanged against PBS using 10 kDa cut-off centrifugal filters, then further purified using size exclusion chromatography (GE, Superdex-200 increase 10/300 GL column). The purity of the recombinant protein was assessed by SDS-PAGE and staining with Coomassie brilliant blue dye and the identity of the protein was confirmed by Liquid Chromatography-Mass Spectrometry (LC-MS). Similarly, the control antigens (PF3D7_1404900, PfMSP7 and PF3D7_0308300 used in this study were produced in an ongoing research project under WACCBIP, University of Ghana's recombinant protein production initiative.

### Immunoblotting

New Zealand rabbits were immunized with the purified rPfATRP protein by Bio Basic, Canada and PfATRP-specific rabbit antibody was purified from the crude sera obtained on day 70 post immunization, as described previously (Boyle et al., 2014). The specificity of α-PfATRP rabbit antibody was confirmed by immunoblotting using 3D7 schizont lysates.

### Schizont Arrest Assays

We performed schizont arrest assays as described previously (Raj et al., 2014). Briefly, 3D7 cultures were subjected to two rounds of sorbitol synchronization at the ring stage (4% parasitemia). The parasites were grown to segmented schizonts and were individually incubated with α-PfATRP rabbit antibody (100 μg/ml) and E64 (10 μM) for 12 h. We scored the effect of egress inhibition by measuring the number of newly formed rings instead of residual schizonts and made comparison with the untreated control.

### Immunofluorescence Staining of Parasites

Smears from synchronized *P. falciparum* 3D7 and NF54 cultures were made on glass slides and fixed in pre-chilled methanol (−20°C). The slides were air-dried and blocked using 3% BSA in PBS overnight at 4°C. The slides were incubated at room temperature (~25°C) for an hour with different antibodies at the following dilutions: α-PfATRP rabbit antibody (1:100); BEI Resources MRA-897A anti-*P. falciparum* apical merozoite antigen-1 (α-PfAMA1) mouse monoclonal antibody (1:100), anti-*P. falciparum* merozoite surface protein 1 (α-PfMSP1) mouse monoclonal antibody (1:100); (Guevara Patiño et al., 1997), anti-*P. falciparum* myosin tail interacting protein (α-PfMTIP) rat antibodies (1:100); (Jones et al., 2006), BEI Resources MRA-316A anti-*Plasmodium falciparum* 48/45-kDa Gamete Surface Protein (Pfs48/45) mouse antibody (1:100); (Miller, L. H. and A. Saul, Personal Communication) and α-Tubulin acetyl transferase 1 (α-TAT 1) mouse antibody (1:10). The details for these marker antibodies have been described in the acknowledgment section. After the incubation period, the slides were washed and incubated with secondary antibodies conjugated with FITC, Alexa Fluor 488 or Alexa Fluor 594. The slides were washed and mounted with VECTASHIELD mounting medium (Burlingame, CA) with 4′, 6′-diamidino-2-phenylindole (DAPI) and were viewed on a fluorescence microscope (Olympus BX-41, Hamamatsu Photonics K.K, Japan). The IFA images captured were processed using the Fiji-Image J software (National Institutes of Health, USA).

In-solution IFA was performed by co-labeling non-permeabilized segmented schizonts with α-merozoite surface protein 1 mouse antibody (PfMSP-1) and α-PfATRP rabbit antibody (1:100) as described previously (Raj et al., 2014). The nulcei were stained with Hoechst (ThermoFisher Scientific) and the labeled parasites were mounted on glass slides using VECTASHIELD mounting medium (Burlingame, CA) and images were captured and processed as described above.

### Human Plasma Samples and Ethical Approval

Ethical approval was obtained from the ethics committees of the Noguchi Memorial Institute for Medical Research, University of Ghana, the Ghana Health Service, Navrongo Health Research Center, and Kintampo Health Research Center and all research was performed in accordance with the prescribed guidelines/regulations. Plasma samples were collected after obtaining informed consent from parents/guardians of the participating children. Blood used in this study for culturing was obtained from healthy donors with informed consent.

The human plasma samples used in this study were obtained from children (2–14 years) resident in Kintampo, Navrongo and Accra and the samples were collected during the rainy seasons between September 2011 and September 2013 (Mensah-Brown et al., 2015, 2017). The ages of children recruited in Kintampo were not significantly different from those in Accra and Navrongo (*P* = 0.10 and *P* = 0.09, respectively); (Mensah-Brown et al., 2019). Also, children recruited in Accra were significantly older than those recruited in Navrongo (*P* = 0.002), (Mensah-Brown et al., 2019). Malaria transmission intensity in the selected sample sites have been measured by the entomological inoculation rates and was highest in Kintampo (>250 infective bites/person per year), followed by Navrongo (<250 infective bites/person per year), and lowest in Accra (<50 infective bites/person per year); (Klinkenberg et al., 2008; Owusu-Agyei et al., 2009; Kasasa et al., 2013).

### Enzyme Linked Immunosorbent Assay

Ten micrograms (10μg) of PfATRP, PF3D7_1404900, PfMSP7 and PF3D7_0308300 soluble recombinant proteins in phosphate buffered saline, pH 7.2 were individually coated between 96-well microtiter plates and incubated overnight at 4°C. The plates were washed thrice with Phosphate Buffered Saline (PBS) containing 0.05% Tween 20 (PBST), and blocked with 3% BSA (Bovine Serum Albumin) in PBS overnight at 4°C. After the washing steps, the plates were incubated with plasma samples (1:50 dilution) from anonymized malaria-infected children for an hour at 37°C. The washing steps were repeated, and the plates were incubated with goat anti-Human IgG (H+L) horseradish peroxidase conjugated Secondary antibody (ThermoFisher Scientific #31410), (1:5000 dilution) at 37°C for an hour. After the incubation period, the plates were washed five times with PBST and PBS, and 3, 3′, 5, 5′-tetramethylbenzidine (TMB) was used to develop the reaction during a 20 min incubation in the dark. Optical Density was read at 450 nm using a VARIOSKAN LUX multi-mode microplate reader (Thermo Fischer Scientific, USA). Malaria-naïve European donor samples were used as experimental controls. The cut-off value for ELISA was calculated based on the readouts for the naïve control.

## Data Availability Statement

All datasets generated for this study are included in the article/Supplementary Material.

## Ethics Statement

The studies involving human participants were reviewed and approved by Ethical approval was obtained from the ethics committees of the Noguchi Memorial Institute for Medical Research, University of Ghana, the Ghana Health Service, Navrongo Health Research Center, and Kintampo Health Research Center and all research was performed in accordance with the prescribed guidelines/regulations. The samples were collected after obtaining informed consent from parents/guardians of the participating children. Blood used in this study for culturing was obtained from healthy donors with informed consent. Written informed consent to participate in this study was provided by the participants' legal guardian/next of kin.

## Author Contributions

EA and GA designed the study with contributions from JR. EA, PI, GO, PN, EQ, LT, MA, RA-D, OA, and HM-B performed experiments, analyzed data, and contributed during the preparation of figures. EA and GA wrote the manuscript.

### Conflict of Interest

The authors declare that the research was conducted in the absence of any commercial or financial relationships that could be construed as a potential conflict of interest.

## Supplementary Material

Figure S1 Homology-based modeling for the prediction of PfATRP structure. 3D-model for the full length PfATRP from **(A,B)** Phyre and I-TASSER protein structure prediction portals.

Figure S2 Codon-optimized gene sequences. PfATRP **(i)** and the control genes, PF3D7_1404900 **(ii)**, MSP7 **(iii)** PF3D7_0308300 **(iv)** provided by BioBasic, Canada and GeneScript, USA.

Figure S3 Expression and purification of recombinant PfATRP. **(A)** PfATRP induced and control lysates were analyzed on SDS-PAGE. Red arrow shows induced band (32 kDa). **(B)** Purification by Ni-NTA chromatography and Size Exclusion Chromatography (SEC) showing the monomeric PfATRP. The protein samples were ran along side with color pre-stained protein standard (P7712S) broad range (11–245 kDa).

Figure S4 Purification of control recombinant proteins. the control antigens, **(A)** PfMSP7 (50 kDa), **(B)** PF3D7_0308300 (40 kDa), and **(C)** PF3D7_1404900 (30 kDa) were expressed and purified to apparent homogeneity. The identities of the control recombinant proteins were confirmed by immunoblotting using α-6xHistidine mouse monoclonal antibody.

Figure S5 Co-immunostaining of anti-PfATRP with surface marker antibodies. Dual IFAs for PfATRP were performed in both asexual and sexual parasite stages using surface marker antibodies. **(A)** Slides were prepared for early/late schizont stage and probed with α-PfATRP rabbit antibody (red); (1:100) and α-PfMSP1 mouse antibody (1:100); (green) which showed that PfATRP does not colocalize with PfMSP1 on the merozoite surface. **(B)** Similarly, dual IFAs were performed for gametocytes using α-PfATRP rabbit antibody (1:100), (green) and α-Pfs48/45 mouse antibody (red); (1:100) which showed that PfATRP is not localized on the surface of gametocytes. White lines on IFA images represent scale bar.

Figure S6 Anti-PfATRP antibody from malaria-exposed children detects recombinant PfATRP. **(i)** Immunoblots showed that the non-reactive control plasma (NHS) did not detect recombinant PfATRP. **(ii–v)** The reactive control plasma from malaria-exposed healthy adults, and the individual immunoreactive pools from children across the three endemic sites detected recombinant PfATRP.

Table S1 The identity of recombinant PfATRP was confirmed by LC-MS analysis. Purified recombinant PfATRP was excised from SDS-PAGE gel and subjected to trypsin digestion followed by LC-MS analysis. The tryptic peptides obtained were blasted against *Plasmodium falciparum* database and the unique peptides, molecular weight, sequence coverage and MS/MS counts obtained confirm the identity of the PfATRP (Q9U0I2).
