# Supplementary figures and images for: Molecular Characterization and Immuno-Reactivity Patterns of a Novel Plasmodium falciparum Armadillo-Type Repeat Protein, PfATRP
Source: Front Cell Infect Microbiol. 2020 Mar 20;10:114. doi: 10.3389/fcimb.2020.00114 (PMC7100384; doi:10.3389/fcimb.2020.00114)

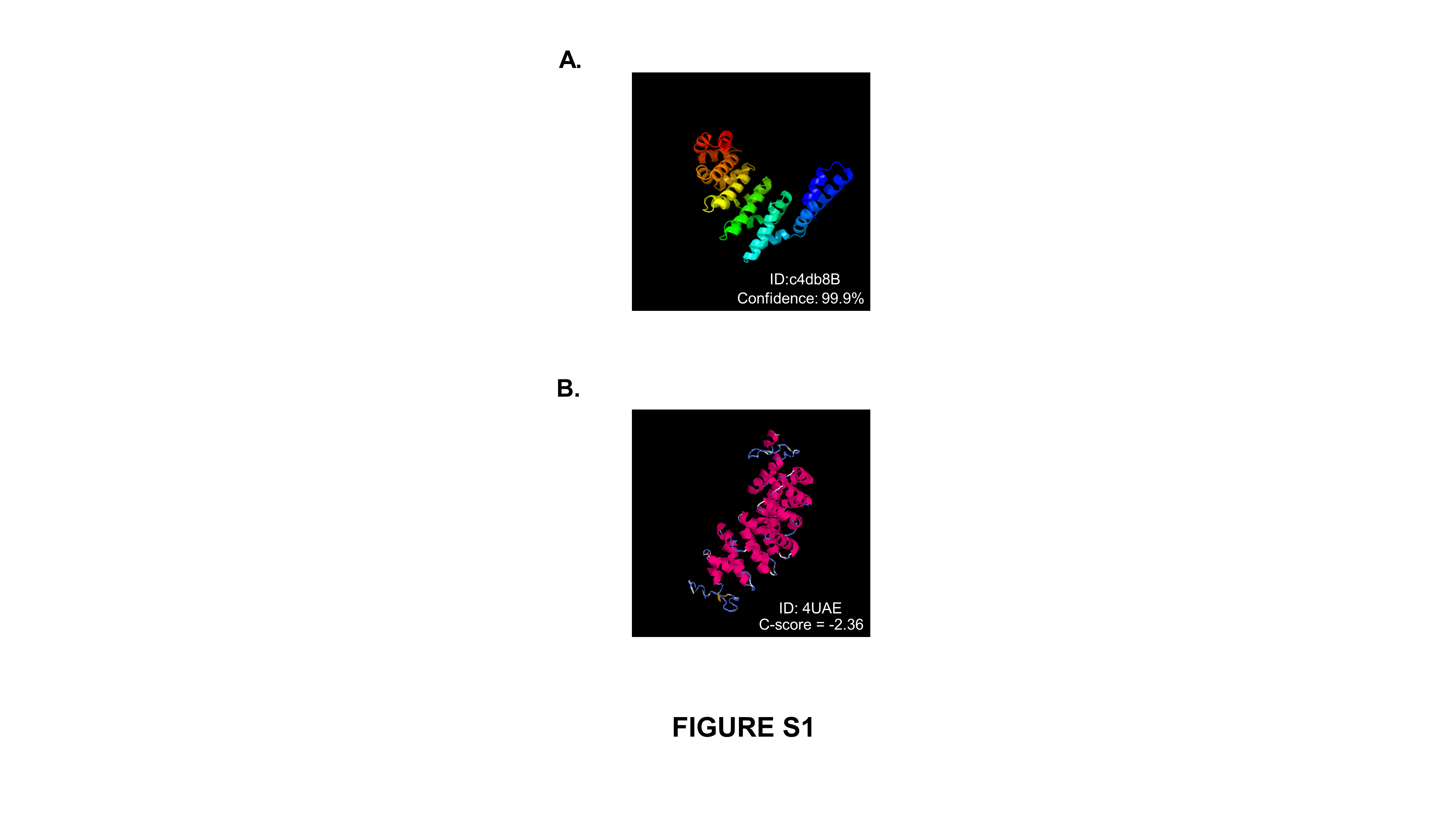

Supplement: Figure S1 — Homology-based modeling for the prediction of PfATRP structure. 3D-model for the full length PfATRP from (A,B) Phyre and I-TASSER protein structure prediction portals. [file Presentation_1.zip › Figure S1.TIF]

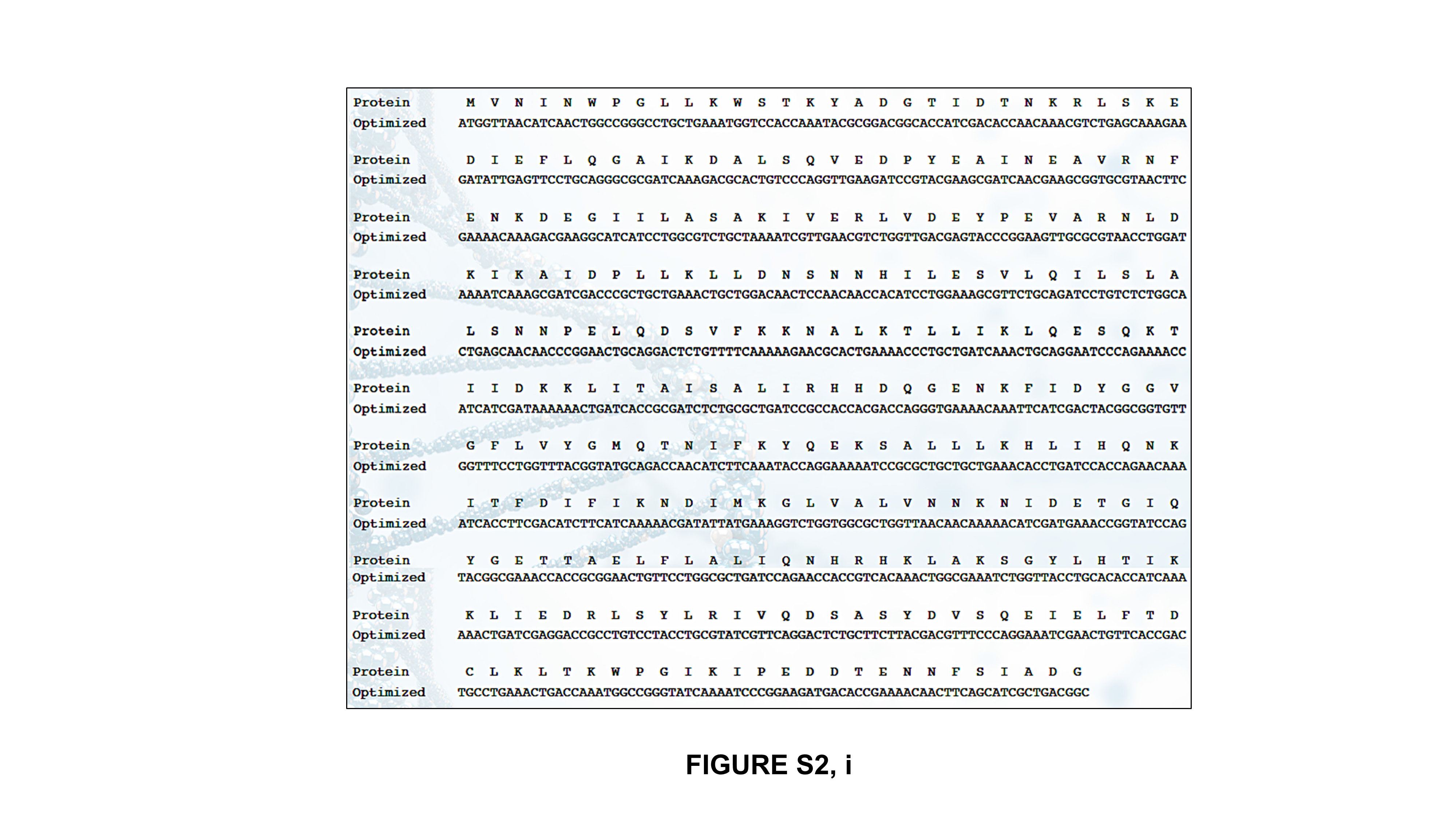

Supplement: Figure S1 — Homology-based modeling for the prediction of PfATRP structure. 3D-model for the full length PfATRP from (A,B) Phyre and I-TASSER protein structure prediction portals. [file Presentation_1.zip › Figure S2i.TIF]

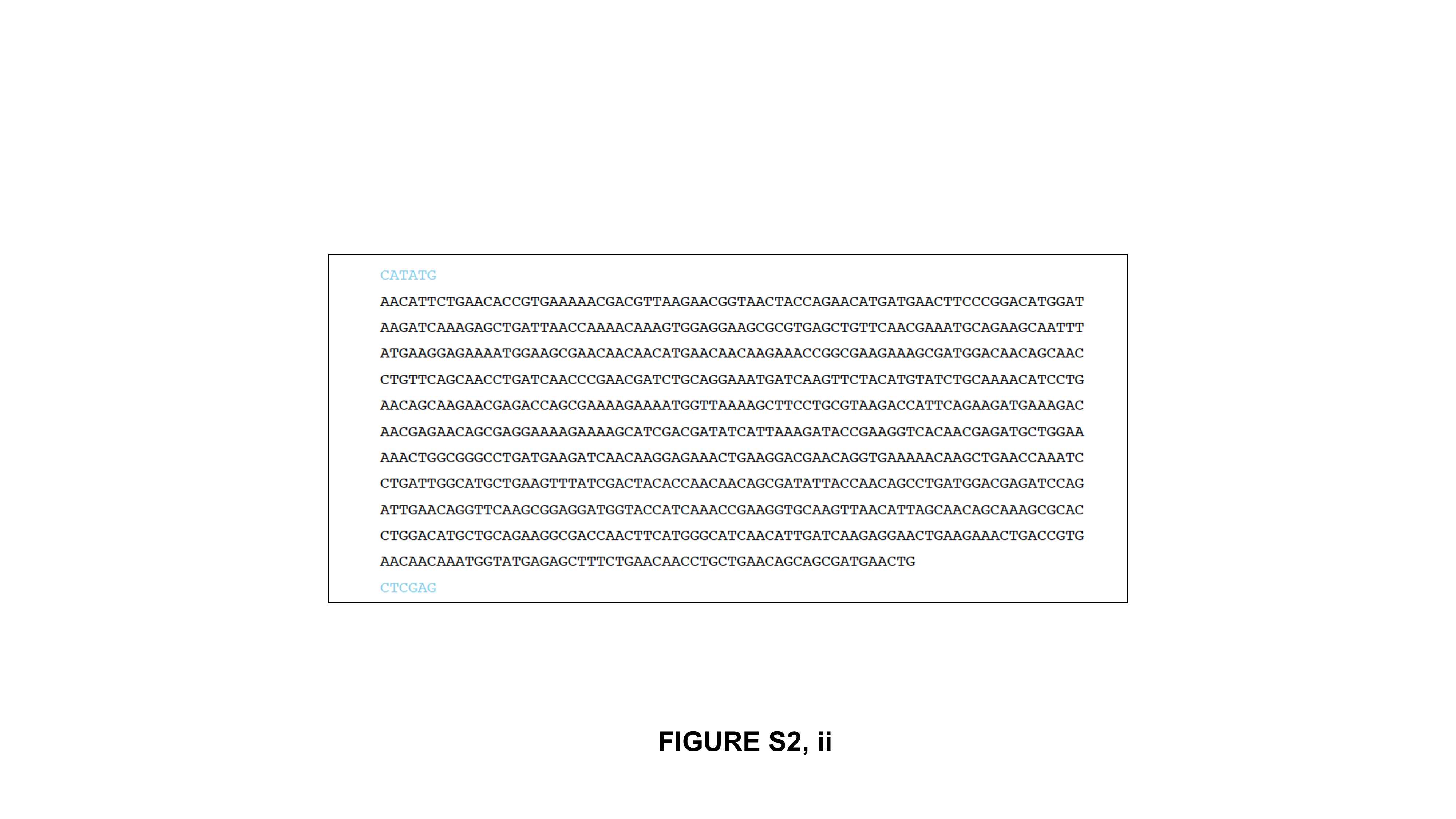

Supplement: Figure S1 — Homology-based modeling for the prediction of PfATRP structure. 3D-model for the full length PfATRP from (A,B) Phyre and I-TASSER protein structure prediction portals. [file Presentation_1.zip › Figure S2ii.TIF]

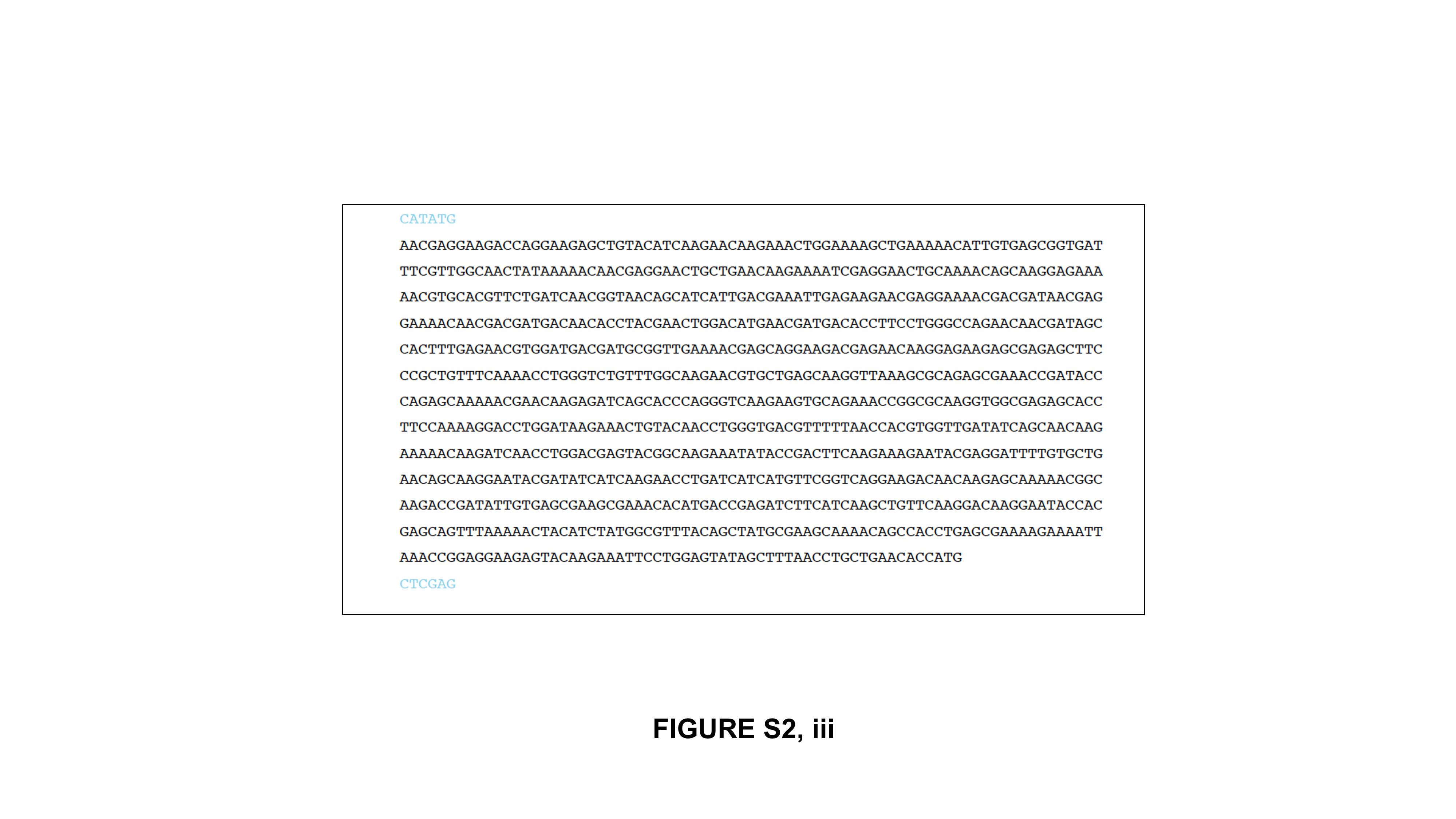

Supplement: Figure S1 — Homology-based modeling for the prediction of PfATRP structure. 3D-model for the full length PfATRP from (A,B) Phyre and I-TASSER protein structure prediction portals. [file Presentation_1.zip › Figure S2iii.TIF]

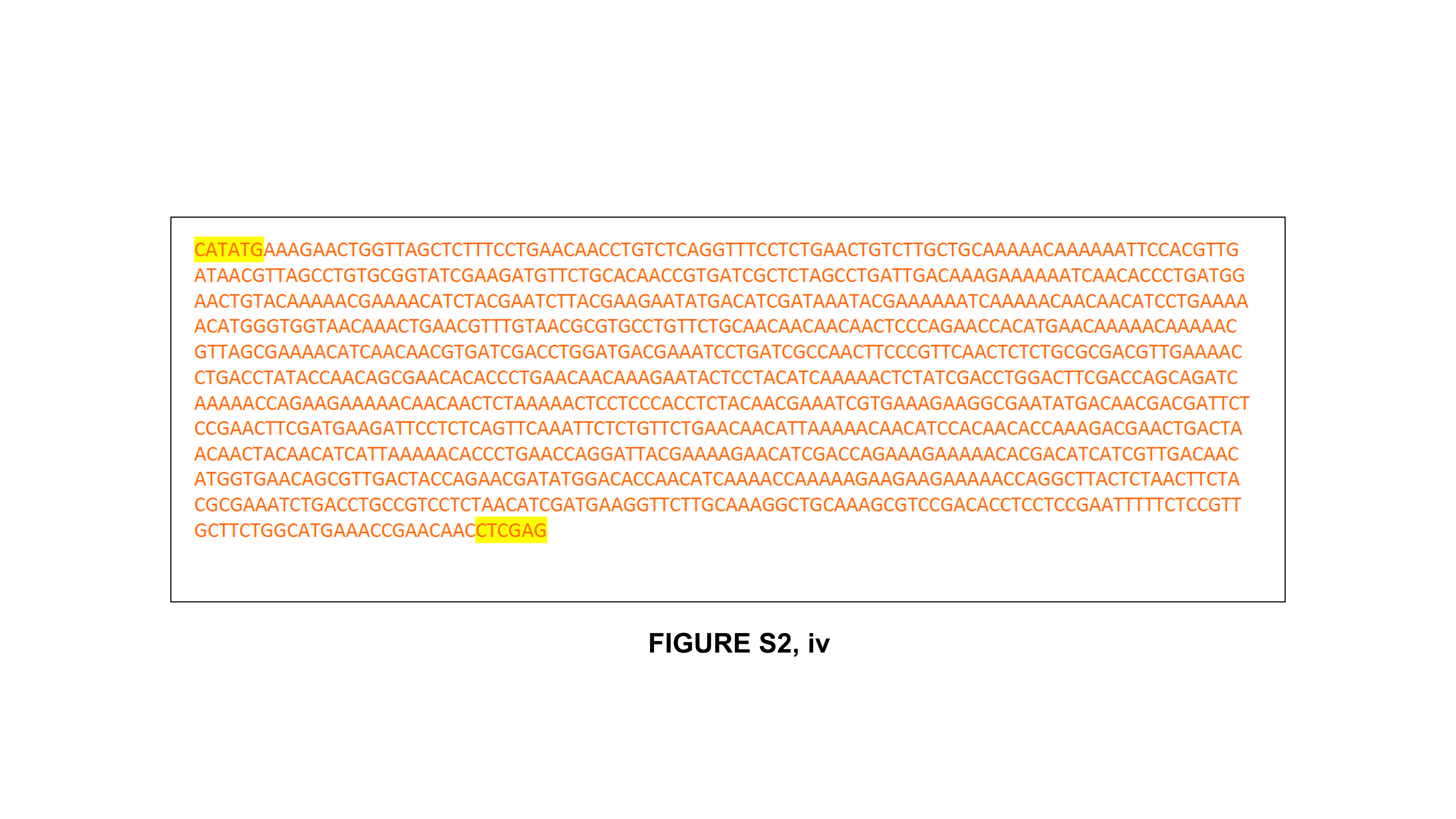

Supplement: Figure S1 — Homology-based modeling for the prediction of PfATRP structure. 3D-model for the full length PfATRP from (A,B) Phyre and I-TASSER protein structure prediction portals. [file Presentation_1.zip › Figure S2iv.TIF]

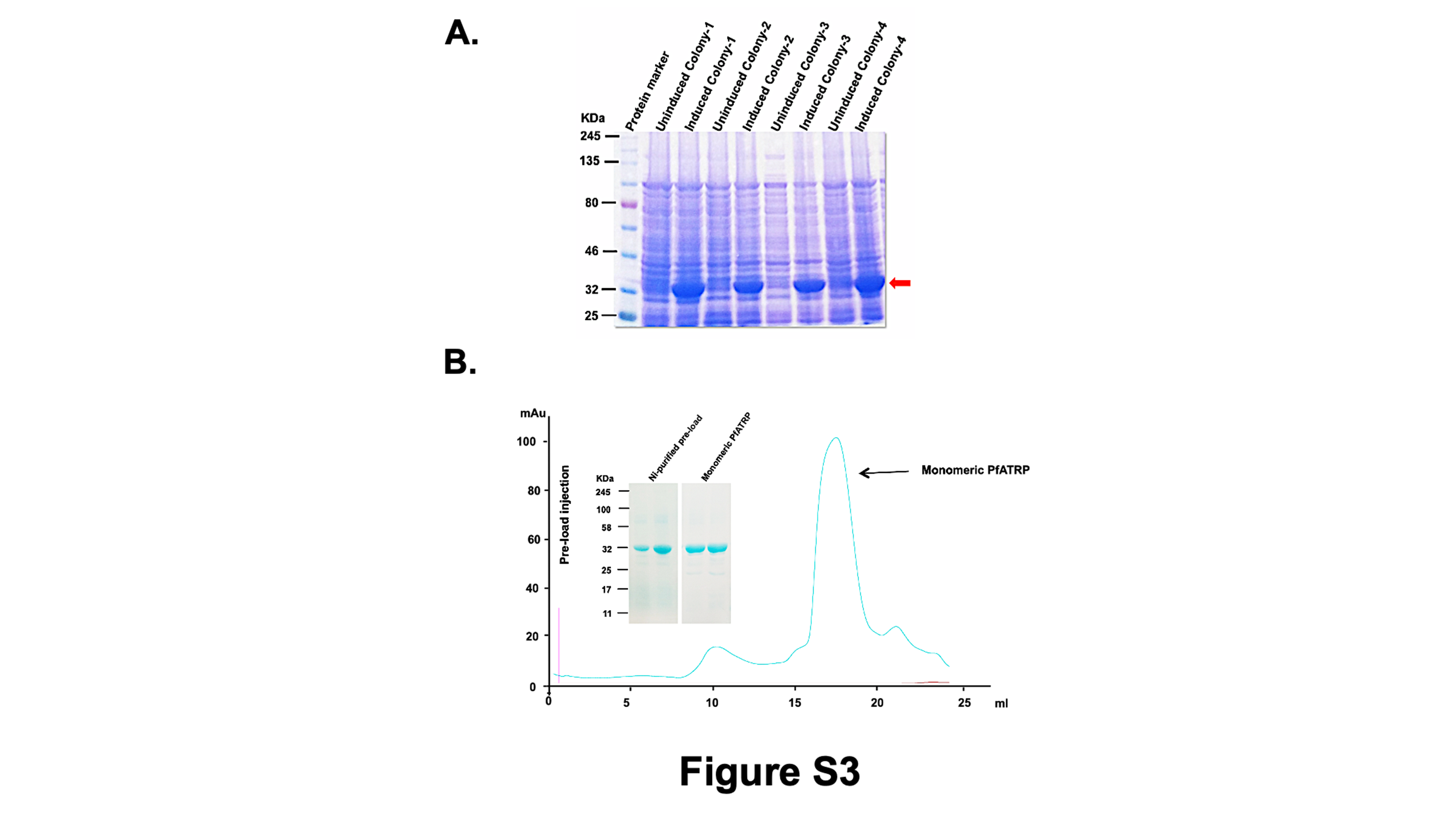

Supplement: Figure S1 — Homology-based modeling for the prediction of PfATRP structure. 3D-model for the full length PfATRP from (A,B) Phyre and I-TASSER protein structure prediction portals. [file Presentation_1.zip › Figure S3.tif]

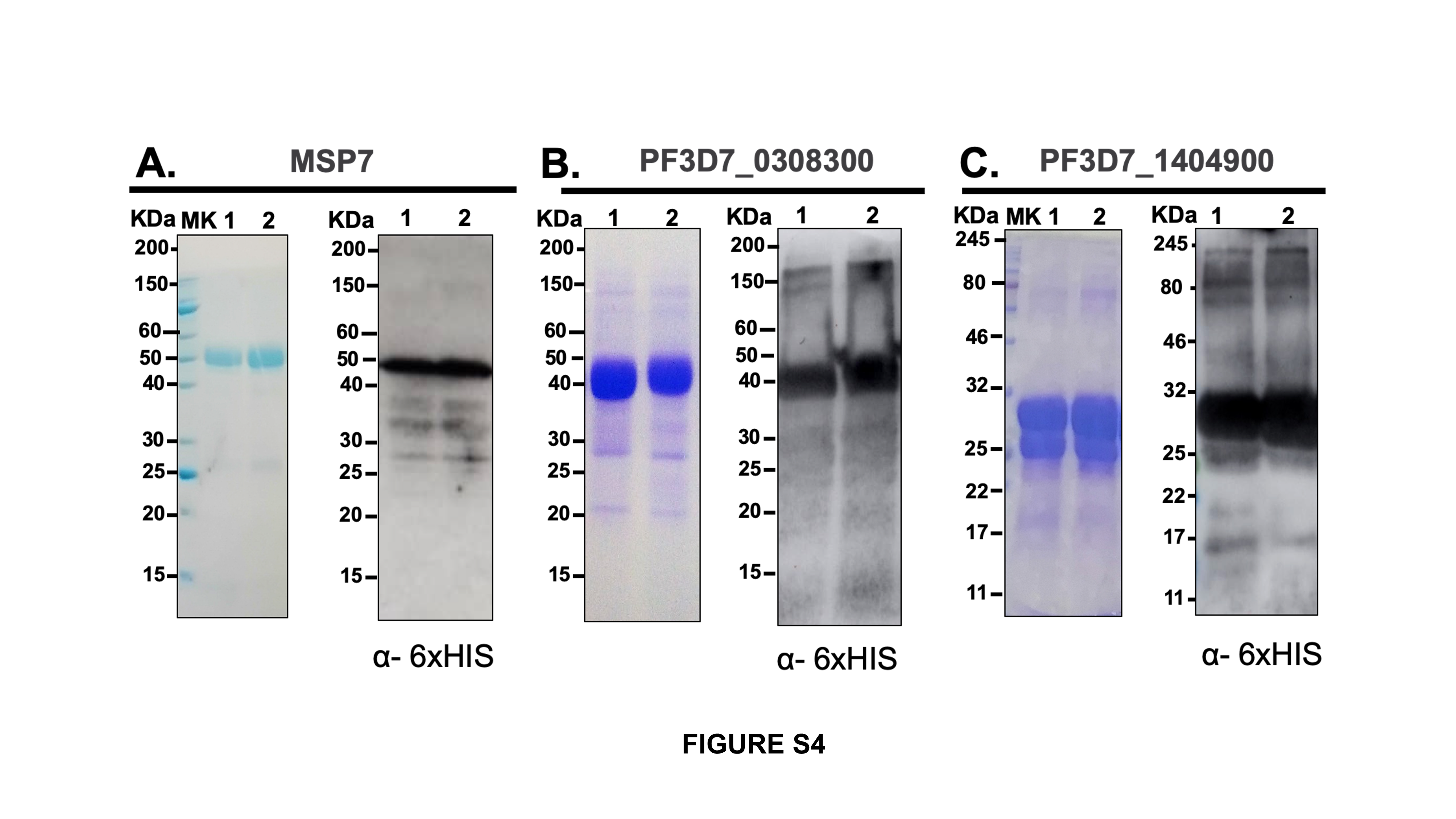

Supplement: Figure S1 — Homology-based modeling for the prediction of PfATRP structure. 3D-model for the full length PfATRP from (A,B) Phyre and I-TASSER protein structure prediction portals. [file Presentation_1.zip › Figure S4.TIF]

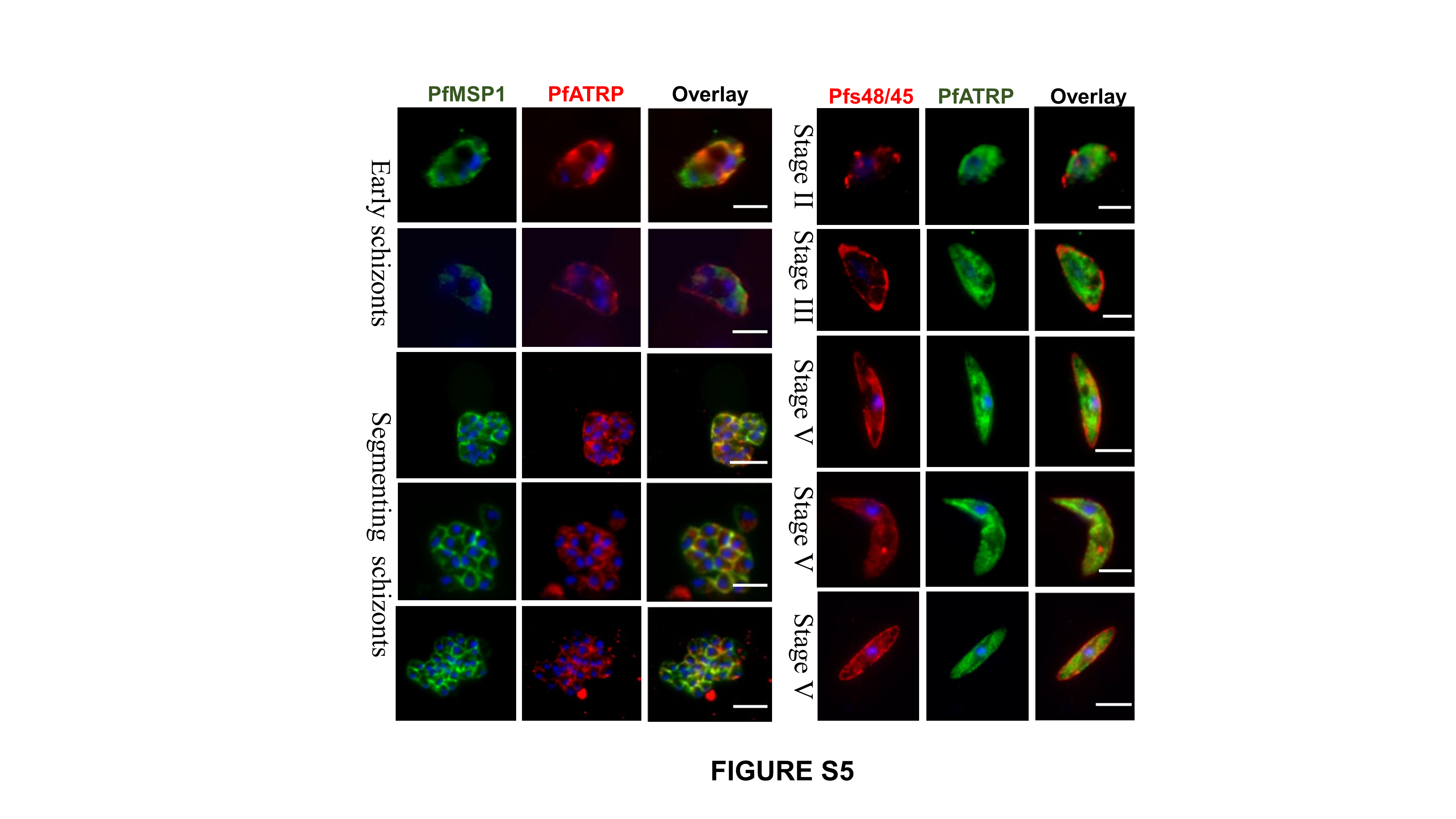

Supplement: Figure S1 — Homology-based modeling for the prediction of PfATRP structure. 3D-model for the full length PfATRP from (A,B) Phyre and I-TASSER protein structure prediction portals. [file Presentation_1.zip › Figure S5.TIF]

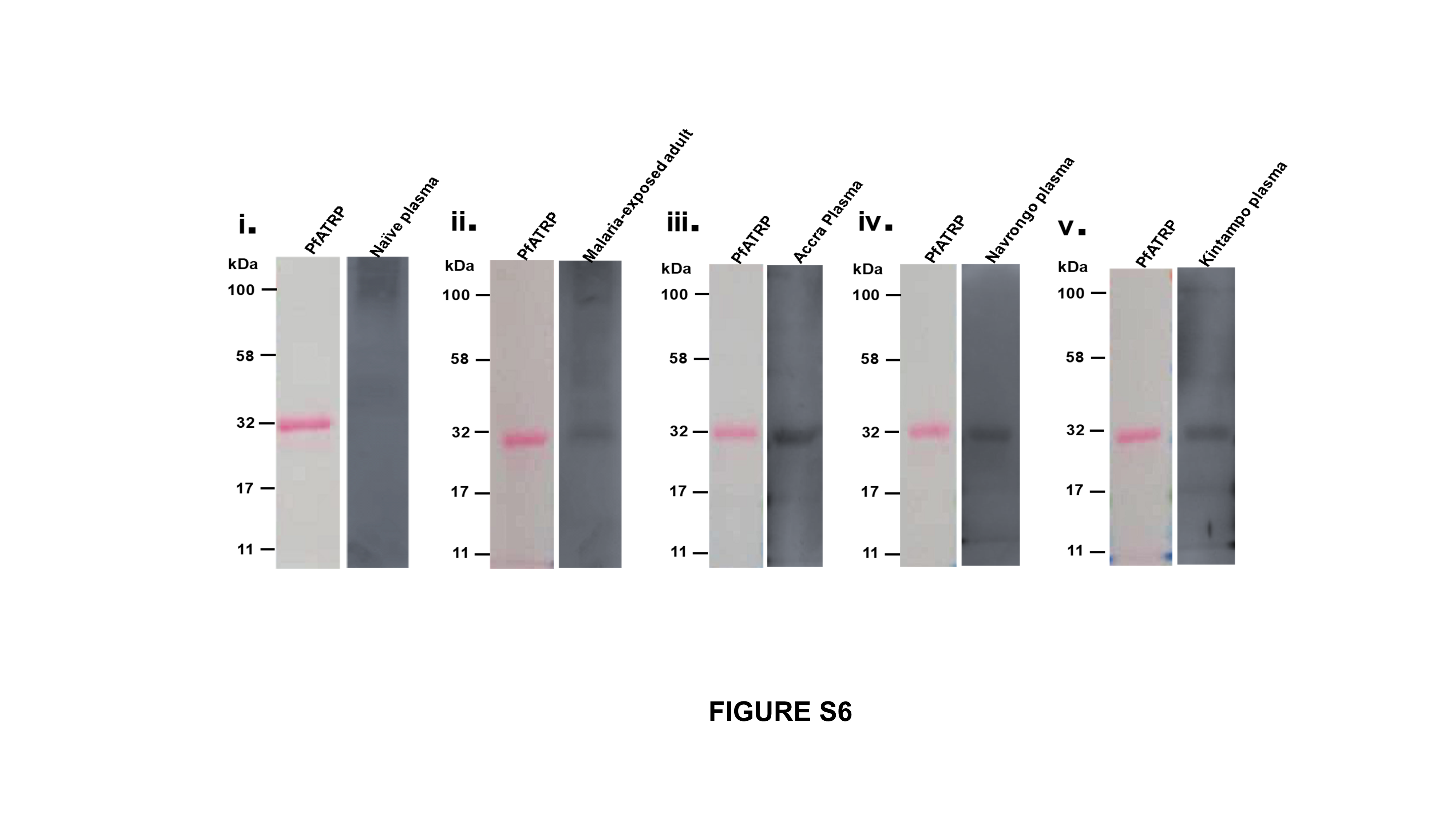

Supplement: Figure S1 — Homology-based modeling for the prediction of PfATRP structure. 3D-model for the full length PfATRP from (A,B) Phyre and I-TASSER protein structure prediction portals. [file Presentation_1.zip › Figure S6.TIF]

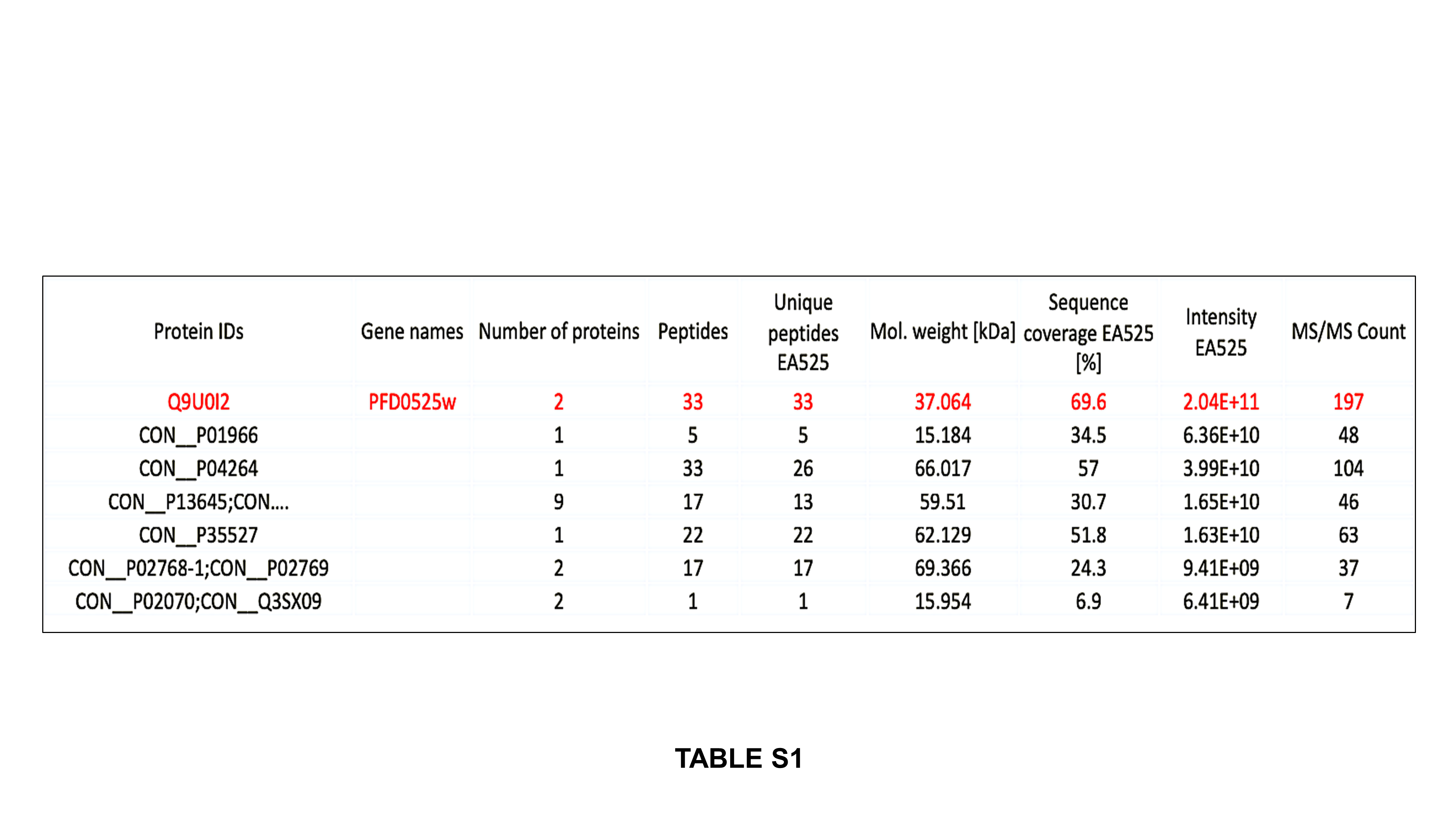

Supplement: Figure S1 — Homology-based modeling for the prediction of PfATRP structure. 3D-model for the full length PfATRP from (A,B) Phyre and I-TASSER protein structure prediction portals. [file Presentation_1.zip › Table S1.TIF]
